# Supplementary material for: The SENTINEL study of differentiated service delivery models for HIV treatment in Malawi, South Africa, and Zambia: research protocol for a prospective cohort study
Source: BMC Health Serv Res. 2023 Aug 23;23:891. doi: 10.1186/s12913-023-09813-w (PMC10463463; doi:10.1186/s12913-023-09813-w)
Supplement: Supplementary file 2 — Additional file 2. [file 12913_2023_9813_MOESM2_ESM.pdf]

D3a. SENTINEL2.0-South Africa Patient Survey - DSD Outcomes

Field	Question	Answer
Screening form		
Screening form > Form control		
screening_no *(required)*	Screening number<br>*screening_no*	
surveyor_id *(required)*	Surveyor ID	
specify_surveyor *(required)*	Specify the surveyor	
district *(required)*	District name	
wr_facilities *(required)*	Facility name	
mp_facilities *(required)*	Facility name	
kzn_facilities *(required)*	Facility name	
screening_date *(required)*	Date	
intro	I. Introduction<br>*Ask the patient for a few minutes of their time. Introduce yourself and the study. Provide information on the study as per the training and give the patient the information sheet for the study. If the patient expresses interest in being in the study, proceed to section II below and then complete the eligibility screening in section III. If the participant is not interested in being in the study, thank them for their time, end the interaction, and answer the questions in section II below.*	
Screening form > II. Demographic description		
gender *(required)*	1. Gender	0 Male<br>1 Female<br>2 Other
age *(required)*	2. How old were you at your last birthday?<br>*Age (years)*	
Screening form > III. Eligibility screening		
Eligibility_note	If all answers to the following screening questions 3-5 and 7 are “YES”, proceed to administer the informed consent process. If any answers are “NO”, thank the patient for their time and end the interaction.	
HIV_care_at_current_facility *(required)*	3. Are you currently enrolled in HIV care at this facility?<br>*STOP if the response is NO!*	1 Yes<br>0 No
is_patient_18 *(required)*	4. Are you currently age 18 or older?<br>*STOP if the response is NO!*	1 Yes<br>0 No
onART_for_6months *(required)*	5. Have you been taking ART for at least 6 months?<br>*STOP if the response is NO!*	1 Yes<br>0 No

Field	Question	Answer
HIV_care_model *(required)*	6. Which HIV care model are you currently enrolled in? (Multiple responses possible for integrated models)<br>*(Note: this question provides the basis for question 5.)*	1 Standard or conventional care, not in an alternative model AND NOT eligible for an alternative model for stable patients<br>2 Standard or conventional care, not in an alternative model AND eligible for an alternative model but not currently enrolled<br>3 Adherence club<br>4 Facility Pick Up Point<br>5 External Pick Up Point<br>6 Youth club<br>7 Pele Box<br>9 Home ART delivery<br>10 Bicycle model<br>8 Other (specify)
other_HIV_care_model *(required)*	Specify the other HIV care model you are currently enrolled in	
collected_medication_once *(required)*	7. Have you had at least one medication collection visit under the model of care you said you are enrolled in?<br>*STOP if the response is NO!*	1 Yes<br>0 No
Screening form > IV. Eligibility decision		
eligible	This patient is ELIGIBLE for the study. Please proceed with the informed consent process	
consenting *(required)*	This patient	1 CONSENTED to participate (Fill in survey instrument ID number below and proceed with survey)<br>2 DID NOT CONSENT to participate (Thank the participant for their time and end the interaction)<br>3 N/A (Target for this model has been reached)
V. Patient survey		
sid *(required)*	Survey ID options	1 Barcode<br>2 Enter manually
barcode_scan *(required)*	Scan survey ID	
survey_id *(required)*	SURVEY ID	
V. Patient survey > Notes		
facility_loc *(required)*	Location within facility	
intro_statement	"Thank you for agreeing to participate in this survey. My name is ______. I will be asking you the questions. Most of the questions require that you select one of the options as your answer, although some questions you can select all the answers that apply. I will specify the options and instructions for you as I ask each question. If your answer is not one of the specified options please tell me and I will write your answer down. Please feel free to tell me whatever you are comfortable sharing. You should also remember that you do not have to share anything that you are not comfortable sharing and that you can stop this interview at any time without any risk to your rights or treatment and care. There are no right or wrong answers, so please be honest and help us to understand what is true for you and your community. Are you ready to begin?<br>*Read the following statement. Please repeat the statement translated into the local language based on primary languages.*	
V. Patient survey > Respondent demographics and socio-economic status		
demographics_statement	"I'm going to start by asking you some basic questions about who you are, where you live, and your education and employment."	
nationality *(required)*	1. What is your nationality/country of origin?	1 South Africa<br>2 Botswana<br>3 Lesotho<br>4 Mozambique<br>5 Malawi<br>6 Namibia<br>7 Swaziland<br>8 Zambia<br>9 Zimbabwe<br>10 Tanzania

Field	Question	Answer
		11 Burundi<br>12 Other African country (specify)<br>13 Other (specify)
other_nationality *(required)*	Please specify nationality/country of origin	
years_in_sa *(required)*	2. If you are not South African, how long have you lived in South Africa?	1 < 1 yr<br>2 1-2 years<br>3 2-5 years<br>4 >5 years<br>5 Seasonal work (Only work in South Africa during certain times of the year)
marital_status *(required)*	3. What is your marital status?	1 Never married<br>2 Married (customary/traditional or legal/civil)<br>3 Divorced<br>4 Separated<br>5 Widowed
have_a_partner *(required)*	4. Is there someone who you have a relationship with and who you call your partner?	1 Yes<br>0 No
living_with_spouse *(required)*	5. Do you currently live with your husband/wife or your partner?	1 No<br>2 Yes, married or living together
current_household *(required)*	6. Do you think of the house you currently live in as your main house?	1 Yes<br>2 No, my main house is somewhere else in South Africa<br>3 No, my main house is in another country
V. Patient survey > Respondent demographics and socio-economic status > Education		
reading_ability *(required)*	7. Do you know how to read and write?	1 No<br>2 Yes – read and write<br>3 Yes – read only
edu_level *(required)*	8. What was the highest level of school that you completed?	1 No schooling<br>2 Primary<br>3 Secondary<br>4 Certificate/Diploma/ Post-secondary<br>5 Graduate degree
occupation *(required)*	9. What is your occupation?	1 Farming (my own or my family's farm)<br>2 Farm worker (someone else's farm)<br>3 Domestic worker or carer (paid)<br>4 Informal sector job (not farming or domestic) (e.g. trader, day service provider)<br>5 Formal sector job (salaried)<br>6 Household work and/or childcare (my own house, not paid)<br>7 Unemployed but looking for work<br>8 Student or trainee<br>9 Retired<br>11 Self-employed/own business<br>12 Unemployed but not looking for work<br>10 Other (specify)
other_occupation *(required)*	Please specify your occupation	

Field	Question	Answer
mostmoney *(required)*	10. Where do you get MOST of your money from?	1 Salary, business or job (formal or informal sector)<br>2 Government social grant<br>3 Spouse/partner<br>4 Parents/relatives<br>5 Friends<br>6 Other (specify)
specifymoney *(required)*	Please specify	
lack_of_food *(required)*	11. Do you or the people in your household go without food often, sometimes, seldom, never?	1 Never<br>2 Seldom<br>3 Sometimes<br>4 Often
government_grant *(required)*	12. Do you or does anybody in your household, currently receive any support or grant from the government?<br>*Tick all that apply*	0 No<br>1 Child grant<br>2 Partial disability / illness grant / temporary grant<br>3 Pension grant<br>4 Disability grant<br>5 Unemployment grant/UIF<br>7 COVID social relief grant<br>6 Other (specify)
other_support_grant *(required)*	Please specify the support or grant from the government	
healthcare_money *(required)*	13. If a person in your household became ill and 100 Rands was needed for treatment or medicines, would you say it would be very easy, easy, difficult, or very difficult to find the money?	1 Very difficult<br>2 Difficult<br>3 Easy<br>4 Very easy
V. Patient survey > Healthcare access and cost		
V. Patient survey > Healthcare access and cost > ART questions		
HIVtreatment_duration *(required)*	14. How long have you been taking ART?<br>*Enter years*	
HIVtreatment_duration_months *(required)*	Surveyor: Now enter the number of months<br>*Months*	
months_for_med *(required)*	15. On average, how many months of ART medication do you receive at a time?<br>*Months*	
additionaldiseases *(required)*	16. Do you have other conditions/illnesses in addition to HIV?	1 Yes<br>0 No
additionalservices *(required)*	17. Which health care services are you routinely receiving at this facility, in addition to HIV care?<br>*(Tick all that apply)*	1 TB<br>2 Diabetes<br>3 Hypertension<br>4 Asthma<br>5 Mental health<br>6 Malaria<br>7 Family planning<br>8 Antenatal care<br>9 Child health care<br>10 TB preventative therapy (TPT)<br>11 Other (specify)
specifyaddservices *(required)*	Please specify	
V. Patient survey > Healthcare access and cost > Follow-up questions ([additionalservices_name]) (1)		(Repeated group)
receivettreatment *(required)*	18. Do you receive treatment for [additionalservices_name] or as part of the health care services you are routinely receiving at this facility?	1 Yes<br>0 No
combinevisits *(required)*	19. Are you able to combine your HIV visits with the visits for [additionalservices_name]?	0 Always<br>1 Very often<br>2 Sometimes<br>3 Rarely<br>4 Never
collectforall *(required)*	20. Are you able to collect your medication for [additionalservices_name] at the same time as you collect your HIV medication?	0 Always<br>1 Very often<br>2 Sometimes<br>3 Rarely<br>4 Never

Field	Question	Answer
healthcare_professional_visited *(required)*	21. In the past 12 months have you sought health care from any other health care provider outside this facility? *Tick all that apply*	0 No<br>1 Hospital<br>2 Private doctor<br>3 Traditional healer<br>4 Community health worker<br>5 Local NGO/FBO<br>6 Other (specify)
other_health_providers_visited *(required)*	Please specify other health care providers you sought health care from any outside this facility	
payforservice *(required)*	22. Did you have to pay for the service?	1 Yes<br>0 No
medicalaid *(required)*	23. Are you covered by Medical Aid, Private health insurance, Medical Benefit Scheme, Provident Scheme, or Hospital Plan that helps you pay for health care or medication?	1 Yes<br>0 No
transport_to_clinic *(required)*	24. How do you usually get to the clinic? *Tick all that apply*	1 Walk<br>2 Mini-bus/common taxi<br>3 Own car<br>4 Meter taxi/Uber/Taxify/Hired taxi<br>5 Brought by family/friends in their vehicles<br>6 Other (specify)
other_transport_to_clinic *(required)*	Please specify other means of getting to the clinic	
V. Patient survey > Healthcare access and cost > Get to clinic		
travel_time *(required)*	25. How long does it take you to get to the clinic? (One way – from home to the clinic) *Enter hours*	
travel_time_minutes *(required)*	Surveyor: Now enter minutes *Minutes*	
otherwisedoing *(required)*	26. What would you otherwise have been doing if you had not come to the clinic today?	1 Housework<br>2 Childcare (own children)<br>3 Caring for a relative or friend<br>4 Voluntary work<br>5 Leisure activities<br>6 Attending school or university<br>7 On sick leave<br>8 Seeking work<br>9 Paid work<br>10 Other (specify)
specifyotherwise *(required)*	Please specify	
expenses_incurred *(required)*	27. What expenses/costs do you incur for each clinic visit?	0 No costs<br>1 Transport<br>2 Loss of income due to missing work<br>3 Child care<br>4 Food/drinks<br>5 Other (specify)
other_expenses *(required)*	Please specify other expenses/costs you incur for each clinic visit	
transport_costs *(required)*	28. Please estimate how much does public transport cost you in Rands each time you visit the clinic (Return trip – to the clinic and back home) *Amount in Rands*	
unpaid_time_costs *(required)*	29. Please estimate how much does unpaid time off work cost you in Rands each time you visit the clinic *Amount in Rands*	
childcarecosts *(required)*	30. Please estimate how much does child care cost you in Rands each time you visit the clinic *Amount in Rands*	
foodcosts *(required)*	31. Please estimate how much food/drinks cost you in Rands each time you visit the clinic *Amount in Rands*	
othercosts *(required)*	32. Please estimate how much does the 'other specified' cost you in Rands each time you visit the clinic *Amount in Rands*	
V. Patient survey > Healthcare access and cost > How many visits		
nurse_and_collection_visit *(required)*	33. For your HIV treatment, how many visits to this clinic where you both see a nurse and collect your medication do you attend per year? *Number of visits per year*	
nurse_and_collection_hours *(required)*	34. How long does it take total, on average for each HIV clinic visit where you see a nurse and pick up medications (counting from when you arrive at the clinic to when you leave)? *Enter hours*	
nurse_and_collection_minutes *(required)*	Surveyor: Now enter minutes	

Field	Question	Answer
	Minutes	
med_collection_visit_only *(required)*	35. For your HIV treatment, how many visits to this clinic where you do NOT see a nurse but do collect your medication do you attend per year?<br>*Number of visits per year*	
med_collection_hours *(required)*	36. How long does it take total, on average for each ART medication pick-up (visits where you only pick up medications, do not see a nurse)?<br>*Enter hours*	
med_collection_minutes *(required)*	Surveyor: Now enter minutes<br>*Minutes*	
missed_visits *(required)*	37. Have there been occasions where you missed your facility visits in the past year by more than 7 days?	1 Yes<br>0 No
missed_visits_no *(required)*	38. How many facility visits have you missed by more than 7 days?<br>*Visits missed in the past year*	
missed_visit_reason *(required)*	39. Thinking of the most recent facility visit you missed, what was the MAIN reason you missed the visit?	1 Forgot pick up date<br>2 Ill health<br>3 Nobody else to go for me<br>4 Buddy forgot<br>5 Buddy unwell<br>6 No money for transport<br>7 Could not leave work<br>8 Afraid HIV status will get known<br>10 Travelling/away from home<br>11 My appointment date/time was no longer convenient<br>9 Other (specify)
other_missed_visit_reasons *(required)*	Please specify other reasons for missing the visit	
stillhaveart *(required)*	40. Did you still have ART medication in hand even though you missed your facility visit?	0 No<br>1 No, but I was able to collect ART medication elsewhere<br>2 Yes, I still had ART medication
specifylocation *(required)*	Specify where you collected the medication	
facilitycall *(required)*	41. Did someone from the facility call/message/visit you after you missed the facility visit?	1 Yes<br>0 No
feelsick *(required)*	42. What do you do if you feel sick or have questions related to your healthcare when you do not have a scheduled visit?	1 Make a special visit to this clinic<br>2 Wait until my scheduled visit to this clinic<br>3 Contact a healthcare provider via phone<br>4 Other (specify)
specifyfeelsick *(required)*	Please specify	
treatmentquestions *(required)*	43. If you have a question about your HIV treatment, what would you most likely do?	1 Make a special visit to this clinic<br>2 Wait and ask during a regular visit to this clinic<br>3 Contact a healthcare provider via phone<br>4 Other (specify)
specifytreatmentquestions *(required)*	Please specify	
covidchanges *(required)*	44. Did you experience any changes in how you received care for your HIV during the COVID pandemic?	1 Yes<br>0 No
whatchanged *(required)*	45. What changed?<br>*(Tick all that apply)*	1 Staff shortages<br>2 Longer waiting times<br>3 Medication stockout<br>4 Clinic was closed for a period of time<br>5 Turned away without receiving services because of the reduced number of patients the facility could attend to because of social distancing

Field	Question	Answer
		6 I received more months of medication<br>7 I received a 12-month script<br>8 Offered a different, more convenient medication collection location<br>9 I was offered home ART delivery<br>10 Other (specify)
specifychange *(required)*	Please specify	
V. Patient survey > Questions for patients in standard care		
ever_enrolled_in_DSD *(required)*	46. Have you ever been enrolled in a DSD model such as Adherence Club, Ex-PuP, Fac-PuP and etc.?	0 No<br>1 Yes<br>2 Don't know
which_model *(required)*	47. Which DSD model were you enrolled in?	1 Adherence club<br>2 Facility Pick Up Point<br>3 External Pick Up Point<br>4 Youth club<br>5 Pele Box<br>7 Home ART delivery<br>8 Bicycle model<br>9 Dablap<br>10 Sha'p left<br>6 Other
specify_model *(required)*	Please specify the model you were enrolled in.	
back_referral_reasons *(required)*	48. Can you recall the reasons you were referred back to regular (standard of care) HIV services by the model's staff?<br>*(Tick all that apply)*	0 No<br>1 Ill health<br>2 Missed a visit<br>3 Missed ARV doses<br>4 Blood draw<br>5 Screened positive for TB<br>6 Diabetes complication<br>7 Hypertension complication<br>8 Don't know why<br>9 Other
specifybackreferral *(required)*	Please specify	
notenrolled *(required)*	49. What is the reason you are currently not enrolled in a DSD model?	1 I prefer standard of care (attending normal clinic visits)<br>2 The clinic is more convenient for me<br>3 I was never offered/ given a choice<br>4 I have another condition for which I need to come to the clinic (specify)<br>5 I don't know<br>6 Other (specify)
specifynotenrolled *(required)*	Please specify	
V. Patient survey > Questions for patients in standard care > 50 a. Accessibility /convenience		
labelconvenience	a. Accessibility /convenience (How convenient to you are the following?):	1 very inconvenient<br>2 inconvenient<br>3 neither convenient nor inconvenient<br>4 convenient<br>5 very convenient
refillplace *(required)*	i. Place you go for your drug refill	1 very inconvenient<br>2 inconvenient<br>3 neither convenient nor inconvenient<br>4 convenient<br>5 very convenient

Field	Question	Answer
dayoftheweek *(required)*	ii. Day of the week you are scheduled to pick up your drugs	1 very inconvenient<br>2 inconvenient<br>3 neither convenient nor inconvenient<br>4 convenient<br>5 very convenient
timeoftheday *(required)*	iii. Time of the day you pick up your drugs	1 very inconvenient<br>2 inconvenient<br>3 neither convenient nor inconvenient<br>4 convenient<br>5 very convenient
placeforreview *(required)*	iv. Place for the clinical consultation	1 very inconvenient<br>2 inconvenient<br>3 neither convenient nor inconvenient<br>4 convenient<br>5 very convenient
dayforreview *(required)*	v. Day of the week you are scheduled for your clinical consultation	1 very inconvenient<br>2 inconvenient<br>3 neither convenient nor inconvenient<br>4 convenient<br>5 very convenient
V. Patient survey > Questions for patients in standard care > 50 b. Environment		
howgood	b. Environment (Tell me how good the following at this facility) are:	1 very good<br>2 good<br>3 fair<br>4 poor<br>5 very poor
neatness *(required)*	vi. Neatness and cleanliness	1 very good<br>2 good<br>3 fair<br>4 poor<br>5 very poor
ease *(required)*	vii. Ease of finding where to go (clinic flow)	1 very good<br>2 good<br>3 fair<br>4 poor<br>5 very poor
privacy *(required)*	viii. Privacy	1 very good<br>2 good<br>3 fair<br>4 poor<br>5 very poor
V. Patient survey > Questions for patients in standard care > 50 c. Efficiency		
howsatisfied	c. Efficiency (how satisfied are the following aspects of your HIV care?):	1 very inefficient<br>2 inefficient<br>3 neither efficient nor inefficient<br>4 efficient<br>5 very efficient
waitingtime *(required)*	ix. Waiting time to see a clinician	1 very inefficient<br>2 inefficient<br>3 neither efficient nor inefficient<br>4 efficient<br>5 very efficient
collectiontime *(required)*	x. Waiting time to collect medication	1 very inefficient<br>2 inefficient<br>3 neither efficient nor inefficient<br>4 efficient<br>5 very efficient

Field	Question	Answer
timewithprovider *(required)*	xi. Time spent with health provider	1 very inefficient<br>2 inefficient<br>3 neither efficient nor inefficient<br>4 efficient<br>5 very efficient
costincurred *(required)*	xii. Cost incurred to attend visit	1 very inefficient<br>2 inefficient<br>3 neither efficient nor inefficient<br>4 efficient<br>5 very efficient
V. Patient survey > Questions for patients in standard care > 50 d. Comprehensiveness		
comprehensiveness	d. Comprehensiveness (how comprehensive were the following aspects of your HIV care?):	1 very non-comprehensive<br>2 non-comprehensive<br>3 neither non-comprehensive nor comprehensive<br>4 comprehensive<br>5 very comprehensive
healthedureceipt *(required)*	xiii. Receipt of health education	1 very non-comprehensive<br>2 non-comprehensive<br>3 neither non-comprehensive nor comprehensive<br>4 comprehensive<br>5 very comprehensive
counsellingreceipt *(required)*	xiv. Receipt of counselling	1 very non-comprehensive<br>2 non-comprehensive<br>3 neither non-comprehensive nor comprehensive<br>4 comprehensive<br>5 very comprehensive
visitsrequired *(required)*	xv. The number of visits you are required to attend	1 very non-comprehensive<br>2 non-comprehensive<br>3 neither non-comprehensive nor comprehensive<br>4 comprehensive<br>5 very comprehensive
V. Patient survey > Questions for patients in standard care > 50 e. Humaneness		
humaneness	e. Humaneness (how satisfied are you with the following aspects relate to your interactions with your HIV care health provider?):	1 very dissatisfied<br>2 dissatisfied<br>3 neither satisfied nor dissatisfied<br>4 satisfied<br>5 very satisfied
stafffriendliness *(required)*	xvi. Staff friendliness towards you	1 very dissatisfied<br>2 dissatisfied<br>3 neither satisfied nor dissatisfied<br>4 satisfied<br>5 very satisfied
confidentiality *(required)*	xvii. Confidentiality	1 very dissatisfied<br>2 dissatisfied<br>3 neither satisfied nor dissatisfied<br>4 satisfied<br>5 very satisfied
staffrespectfulness *(required)*	xviii. Respectfulness of staff	1 very dissatisfied<br>2 dissatisfied<br>3 neither satisfied nor dissatisfied<br>4 satisfied<br>5 very satisfied

Field	Question	Answer
staffattentiveness *(required)*	xix. Staff's attentiveness during interaction	1 very dissatisfied<br>2 dissatisfied<br>3 neither satisfied nor dissatisfied<br>4 satisfied<br>5 very satisfied
opportunitytoask *(required)*	xx. Extent to which staff give client opportunity to ask questions	1 very dissatisfied<br>2 dissatisfied<br>3 neither satisfied nor dissatisfied<br>4 satisfied<br>5 very satisfied
decisionmaking *(required)*	xxi. Extent to which staff involved client in decision-making about your healthcare	1 very dissatisfied<br>2 dissatisfied<br>3 neither satisfied nor dissatisfied<br>4 satisfied<br>5 very satisfied
V. Patient survey > Questions for patients in standard care > Satisfaction rating		
overall_satisfaction *(required)*	51. How would you rate your overall satisfaction with the care that you receive at this facility?	1 very dissatisfied<br>2 dissatisfied<br>3 neither satisfied nor dissatisfied<br>4 satisfied<br>5 very satisfied
overall_satisfaction_explained *(required)*	52. Please explain your satisfaction rating above	
reasonabletime *(required)*	53. What do you think would be a reasonable length of time to wait from arriving at the facility before receiving care from a health provider?<br>*Enter hours*	
reasonableminutes *(required)*	Surveyor: Now enter the minutes	
waittoreceivecare *(required)*	54. How long do you usually wait from arriving at the facility to receive care from a health provider?<br>*Enter hours*	
waittoreceivecaremin *(required)*	Surveyor: Now enter the minutes	
disappointed *(required)*	55. Were you disappointed with any aspect of your care at this facility?	
service_improvement *(required)*	56. How could HIV services in this facility be improved?<br>*(select all that apply)*	1 More staff<br>2 More information provided by staff<br>3 Better, more polite, or friendlier nurse and counselor attitude/manner<br>4 Better, more polite, or friendlier reception and admin attitude/manner<br>5 Better location<br>6 Open different days<br>7 Open different times of day<br>8 Open outside of work hours<br>9 Shorter waiting time<br>10 More counselling when there are problems<br>11 More counselling overall<br>12 Less counselling<br>13 Being able to pick up ARVs at different and more convenient sites<br>14 Being able to pick up ARVs at more convenient times<br>15 Being able to have someone else pick up your ARVs<br>16 Having somebody to support you take your ARVs

Field	Question	Answer
		17 Tracing when missed an appointment<br>18 Reminders via phone<br>19 More months of ARVS given at each visit<br>20 Fewer months of ARVS given at each visit<br>21 Better access to a nurse or clinic staff<br>22 Treatment/support for other illnesses (specify)<br>23 Other (specify and elaborate)
other_service_improvements *(required)*	Please specify other ways HIV services can be improved	
V. Patient survey > Questions for patients in DSD models		
first_enrollment *(required)*	57. Can you recall the first time you were enrolled in this model?	1 Yes<br>0 No
when *(required)*	58. When were you first enrolled?	
V. Patient survey > Questions for patients in DSD models > How long on ART		
howlongonart *(required)*	59. How long had you been on ART when you enrolled in this model?<br>*Enter years*	
onartmonths *(required)*	Surveyor: Now enter the number of months	
ask_to_enroll *(required)*	60. Did you ask to be enrolled in this model?	1 Yes<br>0 No
consent_to_enroll *(required)*	61. Did you have to provide consent to be enrolled in this model?	0 No<br>1 Yes – written consent<br>2 Yes – verbal consent<br>99 Not sure/don't know/can't remember
choice_to_enroll *(required)*	62. Were you given a choice about joining this model?	0 No<br>1 Yes<br>99 Not sure/don't know/can't remember
whatwasthechoice *(required)*	63. What was the choice you were given	
whychoosetojoin *(required)*	64. Why did you choose to join this model?	
counsellingwhenjoining *(required)*	65. Do you receive any counselling, education, and/or adherence support before or at the time you joined this model?	1 Yes<br>0 No
describecounselling *(required)*	66. Please describe	
understandthemodel *(required)*	67. When you joined this model, did you understand the differences between your new model and the previous model or the care you received before you joined the model?	1 Yes<br>0 No
whatyouunderstood *(required)*	68. Please explain what you understood about the care you would receive under this model	
happy_to_enroll *(required)*	69. Were you happy to be enrolled in a DSD model?	0 No<br>1 Somewhat<br>2 Yes<br>3 Neither happy nor unhappy (did not care)
discussmodel *(required)*	70. When last did you discuss your model choice with your provider?	1 Never<br>2 When I first enrolled in my current model<br>3 During my last clinical visit<br>4 At my last drug pick-up<br>5 Other (specify)
specifydiscussmodel *(required)*	Please specify	
awareofmodels *(required)*	71. Are you aware of other DSD models currently available at this facility, and what would be your preferred model?	1 Adherence club<br>2 Facility Pick Up Point<br>3 External Pick Up Point<br>4 Youth club<br>5 Pele Box<br>7 Home ART delivery<br>8 Bicycle model<br>9 Dablap<br>10 Sha'p left

Field	Question	Answer
		6 Other
specifyawareofmodels *(required)*	Please specify	
preferredmodelreason *(required)*	72. Please elaborate on the reasons for your preferred model of care	
V. Patient survey > Questions for patients in DSD models > 73 a. Accessibility /convenience		
labelconvenience2	a. Accessibility /convenience (How convenient to you are the following?):	1 very inconvenient<br>2 inconvenient<br>3 neither convenient nor inconvenient<br>4 convenient<br>5 very convenient
refillplace2 *(required)*	i. Place you go for your drug refill	1 very inconvenient<br>2 inconvenient<br>3 neither convenient nor inconvenient<br>4 convenient<br>5 very convenient
dayoftheweek2 *(required)*	ii. Day of the week you are scheduled to pick up your drugs	1 very inconvenient<br>2 inconvenient<br>3 neither convenient nor inconvenient<br>4 convenient<br>5 very convenient
timeoftheday2 *(required)*	iii. Time of the day you pick up your drugs	1 very inconvenient<br>2 inconvenient<br>3 neither convenient nor inconvenient<br>4 convenient<br>5 very convenient
placeforreview2 *(required)*	iv. Place for the clinical consultation	1 very inconvenient<br>2 inconvenient<br>3 neither convenient nor inconvenient<br>4 convenient<br>5 very convenient
dayforreview2 *(required)*	v. Day of the week you are scheduled for your clinical consultation	1 very inconvenient<br>2 inconvenient<br>3 neither convenient nor inconvenient<br>4 convenient<br>5 very convenient
V. Patient survey > Questions for patients in DSD models > 73 b. Environment		
howgood2	b. Environment (Tell me how good the following at this facility) are:	1 very good<br>2 good<br>3 fair<br>4 poor<br>5 very poor
neatness2 *(required)*	vi. Neatness and cleanliness	1 very good<br>2 good<br>3 fair<br>4 poor<br>5 very poor
ease2 *(required)*	vii. Ease of finding where to go (clinic flow)	1 very good<br>2 good<br>3 fair<br>4 poor<br>5 very poor
privacy2 *(required)*	viii. Privacy	1 very good<br>2 good<br>3 fair<br>4 poor<br>5 very poor

5 very poor

Field	Question	Answer
V. Patient survey > Questions for patients in DSD models > 73 c. Efficiency		
howsatisfied2	c. Efficiency (how efficient are the following aspects of your HIV care?):	1 very inefficient<br>2 inefficient<br>3 neither efficient nor inefficient<br>4 efficient<br>5 very efficient
waitingtime2 *(required)*	ix. Waiting time to see a clinician	1 very inefficient<br>2 inefficient<br>3 neither efficient nor inefficient<br>4 efficient<br>5 very efficient
collectiontime2 *(required)*	x. Waiting time to collect medication	1 very inefficient<br>2 inefficient<br>3 neither efficient nor inefficient<br>4 efficient<br>5 very efficient
timewithprovider2 *(required)*	xi. Time spent with health provider	1 very inefficient<br>2 inefficient<br>3 neither efficient nor inefficient<br>4 efficient<br>5 very efficient
costincurred2 *(required)*	xii. Cost incurred to attend visit	1 very inefficient<br>2 inefficient<br>3 neither efficient nor inefficient<br>4 efficient<br>5 very efficient
V. Patient survey > Questions for patients in DSD models > 73 d. Comprehensiveness		
comprehensiveness2	d. Comprehensiveness (how comprehensive were the following aspects related to your HIV care?):	1 very non-comprehensive<br>2 non-comprehensive<br>3 neither non-comprehensive nor comprehensive<br>4 comprehensive<br>5 very comprehensive
healthedureceipt2 *(required)*	xiii. Receipt of health education	1 very non-comprehensive<br>2 non-comprehensive<br>3 neither non-comprehensive nor comprehensive<br>4 comprehensive<br>5 very comprehensive
counsellingreceipt2 *(required)*	xiv. Receipt of counselling	1 very non-comprehensive<br>2 non-comprehensive<br>3 neither non-comprehensive nor comprehensive<br>4 comprehensive<br>5 very comprehensive
visitsrequired2 *(required)*	xv. The number of visits you are required to attend	1 very non-comprehensive<br>2 non-comprehensive<br>3 neither non-comprehensive nor comprehensive<br>4 comprehensive<br>5 very comprehensive
V. Patient survey > Questions for patients in DSD models > 73 e. Humaneness		
humaneness2	e. Humaneness (how satisfied are you with the following aspects related to your interactions with your HIV care health provider?):	1 very dissatisfied<br>2 dissatisfied<br>3 neither satisfied nor dissatisfied<br>4 satisfied<br>5 very satisfied
stafffriendliness2 *(required)*	xv.i Staff friendliness towards you	1 very dissatisfied<br>2 dissatisfied

Field	Question	Answer
		3 neither satisfied nor dissatisfied<br>4 satisfied<br>5 very satisfied
confidentiality2 *(required)*	xvii. Confidentiality	1 very dissatisfied<br>2 dissatisfied<br>3 neither satisfied nor dissatisfied<br>4 satisfied<br>5 very satisfied
staffrespectfulness2 *(required)*	xviii. Respectfulness of staff	1 very dissatisfied<br>2 dissatisfied<br>3 neither satisfied nor dissatisfied<br>4 satisfied<br>5 very satisfied
staffattentiveness2 *(required)*	xix. Staff's attentiveness during interaction	1 very dissatisfied<br>2 dissatisfied<br>3 neither satisfied nor dissatisfied<br>4 satisfied<br>5 very satisfied
opportunitytoask2 *(required)*	xx. Extent to which staff give client opportunity to ask questions	1 very dissatisfied<br>2 dissatisfied<br>3 neither satisfied nor dissatisfied<br>4 satisfied<br>5 very satisfied
decisionmaking2 *(required)*	xxi. Extent to which staff involved client in decision-making about your healthcare	1 very dissatisfied<br>2 dissatisfied<br>3 neither satisfied nor dissatisfied<br>4 satisfied<br>5 very satisfied
V. Patient survey > Questions for patients in DSD models > Satisfaction rating		
satisfaction_before_model *(required)*	74. How would you rate your overall satisfaction with your ART care BEFORE you started this model?	1 Extremely dissatisfied<br>2 Not satisfied<br>3 Neither satisfied nor dissatisfied<br>4 Satisfied<br>5 Very satisfied
satisfaction_during_model *(required)*	75. How would you rate your overall satisfaction with your ART care now that you are in this model?	1 Extremely dissatisfied<br>2 Not satisfied<br>3 Neither satisfied nor dissatisfied<br>4 Satisfied<br>5 Very satisfied
model_satisfaction_rating *(required)*	76. Please explain your satisfaction rating for this model	
reasonabletime_dsd *(required)*	77. What do you think would be a reasonable length of time to wait from arriving at the clinic before receiving care from a health provider?<br>*Enter hours*	
reasonableminutes_dsd *(required)*	Surveyor: Now enter the minutes	
waittoreceivecare_dsd *(required)*	78. How long do you usually wait from arriving at the clinic to receive care from a health provider?<br>*Enter hours*	
waittoreceivecaremin_dsd *(required)*	Surveyor: Now enter the minutes	
q51_skip	79. Do you attend any out-of-facility HIV events as part of your HIV care? By "event" we mean anything involving your treatment that is not at this facility, including club meetings, picking up medications outside the clinic, etc.	1 Yes<br>0 No
out_of_facility_events *(required)*	80. How many out-of-facility HIV events do you attend per year? By "event" we mean anything involving your treatment that is not at this facility, including club meetings, picking up medications outside the clinic, etc.<br>*Number/year*	
transport_to_model_event *(required)*	81. How do you usually get to the out-of-facility model events?<br>*Tick all that apply*	1 Walk<br>2 Mini-bus/common taxi

Field	Question	Answer
		3 Own car<br>4 Meter taxi/Uber/Taxify/Hired taxi<br>5 Brought by family/friends in their vehicles<br>6 Other (specify)
other_travel_to_model_event *(required)*	Please specify other ways you get to the out-of-facility model events	
V. Patient survey > Questions for patients in DSD models > Travel time		
travel_dsd_hours *(required)*	82. How long does it take you to travel to the out-of-facility model events? (One way – from home to the clinic)<br>*Enter hours*	
travel_dsd_minutes *(required)*	Surveyor: Now enter minutes<br>*Minutes*	
otherwisedoing_dsd *(required)*	83. What would you otherwise have been doing if you had not attended out-of-facility model events?	1 Housework<br>2 Childcare (own children)<br>3 Caring for a relative or friend<br>4 Voluntary work<br>5 Leisure activities<br>6 Attending school or university<br>7 On sick leave<br>8 Seeking work<br>9 Paid work<br>10 Other (specify)
specifyotherwisedoing_dsd *(required)*	Please specify	
expenses_incurred_dsd *(required)*	84. What expenses/costs do you incur for each out-of-facility model event?	0 No costs<br>1 Transport<br>2 Loss of income due to missing work<br>3 Child care<br>4 Food/drinks<br>5 Other (specify)
other_expenses_incurred_dsd *(required)*	Please specify other expenses/costs you incur for each out-of-facility model event	
transport_costs_dsd *(required)*	85. Please estimate how much does transport cost you in Rands each time you make an out-of-facility model visit.<br>(Return trip – to the clinic and back home)<br>*Amount in Rands*	
unpaid_time_costs_dsd *(required)*	86. Please estimate how much income (wages or salary) you lose in Rands each time you attend a model event.<br>*Amount in Rands*	
childcarecosts_dsd *(required)*	87. Please estimate how much child care costs you in Rands each time you attend an out-of-facility event<br>*Amount in Rands*	
foodcosts_dsd *(required)*	88. Please estimate how much food/drinks cost you in Rands each time you attend an out-of-facility event<br>*Amount in Rands*	
othercosts_dsd *(required)*	Please estimate how much does the 'other specified' cost you in Rands each time you attend an out-of-facility event<br>*Amount in Rands*	
V. Patient survey > Questions for patients in DSD models > Out of facility subgroup		
dsd_event_hours *(required)*	89. How long does it take total, on average for each out-of-facility model event (Hours and minutes, including travel time and time at event)<br>*Enter hours*	
dsd_event_minutes *(required)*	Surveyor: Now enter minutes<br>*Minutes*	
overall_satisfaction_dsd *(required)*	90. How would you rate your overall satisfaction with your out-of-facility model events?	1 Extremely dissatisfied<br>2 A little dissatisfied<br>3 Neither satisfied nor dissatisfied<br>4 Satisfied<br>5 Very satisfied
explainsatisfaction *(required)*	91. Please explain your satisfaction rating for your out-of-facility model events	
reasonabletime_outoffacility *(required)*	92. What do you think would be a reasonable length of time to wait from arriving at the out-of-facility model event before receiving care from a health provider?<br>*Enter hours*	
reasonableminutes_outoffacility *(required)*	Surveyor: Now enter the minutes	
waittoreceivecare_outoffacility *(required)*	93. How long do you usually wait from arriving at the out-of-facility model event to receive care from a health provider?<br>*Enter hours*	
waittoreceivecaremin_outoffacility *(required)*	Surveyor: Now enter the minutes	

Field	Question	Answer
referred_back *(required)*	94. Have you been referred back to regular (standard of care) HIV services by this model's staff for any reason? *(Tick all that apply)*	0 No<br>1 Ill health<br>2 Missed a visit<br>3 Missed ARV doses<br>4 Blood draw<br>5 Screened positive for TB<br>6 Diabetes complication<br>7 Hypertension complication<br>8 Don't know why<br>9 Other (specify)
specify_referred_back *(required)*	Specify the reason you were referred back	
differentmodel *(required)*	95. Since the first time you were enrolled in a DSD model, have you ever been enrolled in a different model than you are currently?	1 Yes<br>0 No
previousmodels *(required)*	96. Which model/s were you previously enrolled in? *(tick-all that apply)*	1 Adherence club<br>2 Facility Pick Up Point<br>3 External Pick Up Point<br>4 Youth club<br>5 Pele Box<br>7 Home ART delivery<br>8 Bicycle model<br>9 Dablap<br>10 Sha'p left<br>6 Other
specifypreviousmodels *(required)*	Please specify	
whychangemodels *(required)*	97. What was the reason you changed models?	1 Pregnancy<br>2 End of pregnancy or postpartum period<br>3 My age meant I was eligible for a different model for older individuals<br>4 I moved houses, or my place of work changed<br>5 My VL was elevated (I was unwell)<br>6 My VL became suppressed<br>7 I had stopped treatment<br>8 The model is no longer offered at the facility<br>9 I preferred to be in a different model<br>10 Other (specify)
specifywhychangedmodels *(required)*	Please specify	
missed_visits_dsd *(required)*	98. Have there been occasions in the past year where you missed your out-of-facility model event by more than 7 days?	1 Yes<br>0 No
missed_events *(required)*	99. How many out-of-facility model events have you missed by more than 7 days? *Specify number*	
missed_visit_reason_dsd *(required)*	100. Why did you miss the out-of-facility model event?	1 Forgot pick up date<br>2 Ill health<br>3 Nobody else to go for me<br>4 Buddy forgot<br>5 Buddy unwell<br>6 No money for transport<br>7 Could not leave work<br>8 Afraid HIV status will get known<br>10 Travelling/away from home<br>11 My appointment date/time was no longer convenient<br>9 Other (specify)
other_missed_visit_reasons_dsd *(required)*	Please specify	
stillhaveart_dsd *(required)*	101. Did you still have ART medication in hand even though you missed your out-of-facility model event?	0 No

Field	Question	Answer
		1 No, but I was able to collect ART medication elsewhere<br>2 Yes, I still had ART medication
facilitycall_dsd *(required)*	102. Did someone from the facility call/message/visit you after you missed the out-of-facility model event?	1 Yes<br>0 No
feelsick_outoffacility *(required)*	103. What do you do if you feel sick or have questions related to your healthcare when you do not have a scheduled visit?	1 Make a special visit to this clinic<br>2 Wait until my scheduled visit to this clinic<br>3 Wait until my scheduled out-of-facility HIV event<br>4 Contact a healthcare provider via phone<br>5 Other (specify)
specifyfeelsick_outoffacility *(required)*	Please specify	
treatment_questions *(required)*	104. If you have a question about your HIV treatment, what would you most likely do?	1 Make a special visit to this clinic<br>2 Wait and ask during a regular visit to this clinic<br>3 Wait and ask during a regular out-of-facility HIV event<br>5 Contact a healthcare provider via phone<br>4 Other (specify)
other_treatment_questions *(required)*	Please specify what else you would do if you had a question about your treatment	
contactfacility *(required)*	105. In the past 12 months, if you had questions regarding your visit or medication collection scheduling or location were you able to contact the facility or DSD model service provider by phone?	1 Yes<br>0 No
unabletocontact *(required)*	106. What was the reason?	0 I did not have airtime or data<br>1 I did not have facility contact details<br>2 I did not have the DSD service provider contact details<br>3 The facility contact details were not working<br>4 The DSD service provider contact details were not functional<br>5 The phone call to the facility was not answered<br>6 The phone call to the DSD service provider was not answered<br>7 Other (specify)
specifyunabletocontact *(required)*	Please specify	
receiveinfo *(required)*	107. Did you receive adequate information regarding your clinic visits or medication collection scheduling or location?	0 No<br>1 Yes<br>2 Partly
specifynoinfo *(required)*	Specify	
V. Patient survey > Questions for patients in DSD models > Questions for likert_dsd2		
dsd2_likert	108. Please state how much you agree or disagree with the following statements	1 Strongly disagree<br>2 Mildly disagree<br>3 Neither agree or nor disagree<br>4 Mildly agree<br>5 Strongly agree<br>6 No response/don't know
transport_concerns *(required)*	a. I am concerned about transport cost to get to the clinic	1 Strongly disagree<br>2 Mildly disagree<br>3 Neither agree or nor disagree<br>4 Mildly agree<br>5 Strongly agree<br>6 No response/don't know

Field	Question	Answer
transport_concerns_dsd *(required)*	b. I am concerned about transport cost to get to my model events (e.g. club meetings, medication pickups)	1 Strongly disagree<br>2 Mildly disagree<br>3 Neither agree or nor disagree<br>4 Mildly agree<br>5 Strongly agree<br>6 No response/don't know
unsure_of_dsd *(required)*	c. I am unsure about how the DSD model works	1 Strongly disagree<br>2 Mildly disagree<br>3 Neither agree or nor disagree<br>4 Mildly agree<br>5 Strongly agree<br>6 No response/don't know
hiv_info_insufficient *(required)*	d. I don't receive enough information about HIV and ART	1 Strongly disagree<br>2 Mildly disagree<br>3 Neither agree or nor disagree<br>4 Mildly agree<br>5 Strongly agree<br>6 No response/don't know
long_waiting_time *(required)*	e. I have to wait for a long time to receive care	1 Strongly disagree<br>2 Mildly disagree<br>3 Neither agree or nor disagree<br>4 Mildly agree<br>5 Strongly agree<br>6 No response/don't know
safe_arv_storing *(required)*	f. I am concerned about safely carrying and storing ARVs at home	1 Strongly disagree<br>2 Mildly disagree<br>3 Neither agree or nor disagree<br>4 Mildly agree<br>5 Strongly agree<br>6 No response/don't know
status_disclosure_concerns *(required)*	g. I am concerned about other people finding out that I am HIV positive	1 Strongly disagree<br>2 Mildly disagree<br>3 Neither agree or nor disagree<br>4 Mildly agree<br>5 Strongly agree<br>6 No response/don't know
other_patient_interactions *(required)*	h. I am concerned about having to interact with other patients in my treatment model	1 Strongly disagree<br>2 Mildly disagree<br>3 Neither agree or nor disagree<br>4 Mildly agree<br>5 Strongly agree<br>6 No response/don't know
model_quality_care *(required)*	i. I am concerned about the quality of care in my treatment model	1 Strongly disagree<br>2 Mildly disagree<br>3 Neither agree or nor disagree<br>4 Mildly agree<br>5 Strongly agree<br>6 No response/don't know
no_concerns *(required)*	j. I have no concerns about my HIV treatment or model of service delivery	1 Strongly disagree<br>2 Mildly disagree<br>3 Neither agree or nor disagree<br>4 Mildly agree<br>5 Strongly agree<br>6 No response/don't know
work_troubles *(required)*	k. I have trouble taking time off work to get to clinic visits	1 Strongly disagree<br>2 Mildly disagree<br>3 Neither agree or nor disagree<br>4 Mildly agree<br>5 Strongly agree<br>6 No response/don't know

Field	Question	Answer
work_troubles_dsd *(required)*	l. I have trouble taking time off work to get to model events	1 Strongly disagree<br>2 Mildly disagree<br>3 Neither agree or nor disagree<br>4 Mildly agree<br>5 Strongly agree<br>6 No response/don't know
prefer_few_visits *(required)*	m. I prefer to come to the clinic as few times per year as possible	1 Strongly disagree<br>2 Mildly disagree<br>3 Neither agree or nor disagree<br>4 Mildly agree<br>5 Strongly agree<br>6 No response/don't know
patient_connections *(required)*	n. I like being able to connect with other HIV-positive patients at the clinic	1 Strongly disagree<br>2 Mildly disagree<br>3 Neither agree or nor disagree<br>4 Mildly agree<br>5 Strongly agree<br>6 No response/don't know
patient_connections_dsd *(required)*	o. I like being able to connect with other HIV-positive patients in my model of care	1 Strongly disagree<br>2 Mildly disagree<br>3 Neither agree or nor disagree<br>4 Mildly agree<br>5 Strongly agree<br>6 No response/don't know
otherconcerns *(required)*	p. Do you have other concerns about your HIV treatment or model of treatment delivery	1 Yes<br>0 No
specifyconcern *(required)*	Please specify	
V. Patient survey > Questions for patients in DSD models > Recommendation subgroup		
dsd_recommendation *(required)*	109. Would you recommend this model to another patient who is on ART treatment?	1 Yes<br>0 No
explain_recommendation *(required)*	110. Why or why not?	
disappointed_dsd *(required)*	111. Were you disappointed with any aspect of your care in this DSD model?	
dsd_improvement *(required)*	112. How could services on this model be improved?<br>*(select all that apply)*	1 More staff<br>2 More information provided by staff<br>3 Better, more polite friendlier, nurse and counselor attitude<br>4 Better, more polite, or friendlier, reception and admin staff attitude<br>5 Better location for model events<br>6 Events on different days<br>7 Events at different times of day<br>8 Events outside of work hours<br>9 Shorter waiting time<br>10 More counselling when there are problems<br>11 More counselling overall<br>12 Less counselling<br>13 Being able to pick up ARVs at different and more convenient sites<br>14 Being able to pick up ARVs at more convenient times<br>15 Being able to have someone else pick up your ARVs<br>16 Having somebody to support you take your ARVs

Field	Question	Answer
		17 Contacting you when you miss an appointment<br>18 Reminders via phone or SMS<br>19 More months of ARVs given at each visit<br>20 Fewer months of ARVs given at each visit<br>21 Better access to a nurse or clinic staff<br>22 Treatment/support for other illnesses (specify)<br>23 Other (specify and elaborate)
other_dsd_improvement *(required)*	Specify other ways services on this model could be improved	
V. Patient survey > Questions for patients in DSD models > Conclusion subgroup		
best_dsd_thing *(required)*	113. In conclusion, what is the best thing for you about this model?	
worst_dsd_thing *(required)*	114. And what is the worst thing for you about model?	
closing	Please thank the participant for their time and ask if they have any additional questions about the study.	
notes *(required)*	Surveyor notes	
sid_2 *(required)*	Survey ID options	1 Barcode<br>2 Enter manually
barcode_scan_2 *(required)*	Scan survey ID	
survey_id_repeat *(required)*	SURVEY ID	
not_eligible	This patient is NOT ELIGIBLE for the study. Thank the participant for their time but do not proceed with the survey	
surveyor_initials *(required)*	Surveyor initials	
