# Supplementary material for: The SENTINEL study of differentiated service delivery models for HIV treatment in Malawi, South Africa, and Zambia: research protocol for a prospective cohort study
Source: BMC Health Serv Res. 2023 Aug 23;23:891. doi: 10.1186/s12913-023-09813-w (PMC10463463; doi:10.1186/s12913-023-09813-w)
Supplement: Supplementary file 6 — Additional file 6. [file 12913_2023_9813_MOESM6_ESM.pdf]

D1. SENTINEL2.0-South Africa Time-Motion Form

Field	Question	Answer
Form control		
observer_id *(required)*	Observer ID	
specify_observer *(required)*	Specify the observer	
district *(required)*	District name	
wr_facilities *(required)*	Facility name	
mp_facilities *(required)*	Facility name	
kzn_facilities *(required)*	Facility name	
Observation_day *(required)*	Observer: Please select observation day for this participant	1 Day 1<br>2 Day 2
sid *(required)*	Participant ID options	1 Barcode<br>2 Enter manually
barcode_scan *(required)*	Scan participant ID	
participant_id *(required)*	Participant ID	
cadre *(required)*	Participant cadre	1 Professional Nurse<br>2 Enrolled nurse<br>3 Doctor<br>4 Pharmacist<br>5 Pharmacist Assistant<br>6 Other
other_cadre	Specify other cadre	
observation_date *(required)*	Date	
observation_day *(required)*	Day of week<br>*NB: Please ensure that you select the same day as indicated in the calendar*	1 Monday<br>2 Tuesday<br>3 Wednesday<br>4 Thursday<br>5 Friday<br>6 Saturday<br>7 Sunday

Field	Question	Answer
start_time *(required)*	Time observation started for day	
Participant demographic information		
Participant demographic information > Age and gender		
age *(required)*	1. What is your age in years?	
gender *(required)*	2. What is your gender?	1 Male<br>2 Female
participated *(required)*	3. Have you participated in the provider survey?	1 Yes<br>2 No
providersurveyid *(required)*	Observer please fill in the provider Survey ID<br>*if the participant was enrolled in the provider survey (Domain 2), Please provide the survey ID below*	
Participant demographic information > roleandexperiences		
earliertimeandmotion *(required)*	4. Have you participated in earlier rounds of the Time and Motion study?	1 Yes<br>2 No
currentrole *(required)*	5. What is your current role at this facility?	
yearsinrole *(required)*	6. How many years have you worked in your current role/capacity?<br>*Enter years*	
monthsinrole *(required)*	Observer: Now enter the number of months	
yearsatfacility *(required)*	7. How many years have you worked at this facility?	
monthsatfacility *(required)*	Observer: Now enter the number of months	
Participant demographic information > summaryofworkdays		
hivservicestime *(required)*	8. In a typical five-day work week, how much of your work time do you spend on HIV-related service delivery?<br>*(Number of days, decimal allowed). Enter a zero if no time is spent on HIV-related service delivery*	
hivservicestreatment *(required)*	9. In a typical five-day work week, how much of your work time do you spend providing HIV treatment?<br>*(Number of days, decimal allowed). Enter a zero if no time is spent on providing HIV treatment*	
Participant demographic information > facilityprocedures		
target *(required)*	10. Is there a daily/weekly/monthly target for the number of patients each nurse/counsellor/others should see in a day/week/month?	1 Yes<br>2 No
target_by *(required)*	Observer please fill in the target type (duration)<br>*Please indicate if target is per day, week, or month*	
target_number *(required)*	Observer please fill in the target<br>*Please indicate the target (e.g number of patient)*	
practice_normalpatients *(required)*	11. If there are fewer than normal patients at the facility what is normal practice for clinic staff?	1 a) Staff assigned to other non-patient activities/duties (normal work hours apply)<br>2 b) Staff have more unallocated time but normal work hours apply<br>3 c) Staff can leave once all their patients have been seen and paperwork complete<br>4 d) Other specify
otherpractice_normalpatients *(required)*	Observer please specify other	
practice_fewpatients *(required)*	12. If there are more patients than normally expected what is the normal practice?	1 a) The clinic remains open until all patients are seen (clinic hours extended)<br>2 b) Some patients may be asked to return another day unless urgent (normal clinic hours apply)<br>3 c) Other specify
otherpractice_fewpatients *(required)*	Observer please specify other	
totpatientsseen *(required)*	Total number of unique patients seen by provider today (day of observation)<br>*Please look for this number in the register at the end of the day. Or manually count patients seen by the provider at the end of the observation day*	
source *(required)*	Source<br>*Please specify where you verified the number*	
intro	Introduction<br>*Confirm that the provider has consented to being observed and ask if there are any remaining questions or concerns before you start.*	
Observation of time block (1)		(Repeated group)
start *(required)*	Time observation started	
type *(required)*	Patient time block or non-patient time block	1 a. patient interaction<br>2 b. non-patient interaction
patient *(required)*	Patient's primary category	1 1. Non-HIV (chronic or acute)<br>2 2. HIV not yet on ART

Field	Question	Answer
		3 3. Initiating ART today<br>4 4. First six months on ART<br>5 5. Established on ART > 6 months<br>6 6. Elevated Viral Load<br>7 7. Patient representative or buddy (not patient)<br>8 8. Other (specify)
other_patient_category	Specify other patient category	
visit *(required)*	Primary reason for visit	1 a. Acute emergency or COVID related<br>2 b. Scheduled clinical consultation<br>3 c. Unscheduled clinical consultation<br>4 d. Medication refill or collection only<br>5 e. HIV testing<br>6 f. Other (specify)
other_visit	Specify other reason for visit	
visit_procedure *(required)*	Procedures conducted during visit	1 1. Medication refill or collection<br>2 2. ART initiation<br>3 3. Counseling<br>4 4. Laboratory test or health screening<br>5 5. Re-scripting<br>6 6. Other (specify)
other_visit_procedure *(required)*	Specify other procedure conducted during visit	
model *(required)*	Model patient is enrolled in	1 1. Standard care (not enrolled in DSD models)<br>2 2. Adherence club<br>3 3. Facility pick up point<br>4 4. External pickup point<br>5 5. Youth club<br>6 6. Pele Box or locker<br>7 7. Home ART delivery<br>8 8. Bicycle model<br>9 9. Not applicable (non- ART)<br>10 10. DSD model unknown<br>11 11. Unknown whether in DSD model<br>12 12. Other (specify)
other_model	Specify other model patient is enrolled in	
block *(required)*	Reason for non-patient time block	1 a. Patient related task (completing record, referral, etc.)<br>2 b. DSD model related task (Specify model and activity e.g. Home delivery-preparing medications for delivery, CAG-leading a group)<br>3 c. General administration or meetings<br>4 d. External outreach (community activities)<br>5 e. Personal break (lunch, tea, other personal time)<br>6 f. Training<br>7 g. Free time/ no patients<br>9 h. Transit<br>8 i. Other (specify)
other_block	Specify main reason for other block	

Field	Question	Answer
specify_task *(required)*	Specify the DSD model and related task	
alreadyseen *(required)*	Was this patient already seen by the provider?	1 Yes<br>2 No
observercomments *(required)*	Observer's comments (if any)<br>*Your comments for this time block*	
provider_comments *(required)*	Provider's comments (if any)	
observercomments2 *(required)*	Observer's comments (if any)<br>*Your comments for this time block*	
end *(required)*	Time observation ended	
observer_notes *(required)*	Observer's notes<br>*Your overall comments for the entire day's observation*	
end_time *(required)*	Time observations ended for day	
closing	Please thank the participant for their time and ask if they have any additional questions about the study.	
sid_2 *(required)*	Participant ID options	1 Barcode<br>2 Enter manually
barcode_scan_2 *(required)*	Scan participant ID	
participant_id_repeat *(required)*	PARTICIPANT ID	
